# Understanding the Melanocyte Distribution in Human Epidermis: An Agent-Based Computational Model Approach

**DOI:** 10.1371/journal.pone.0040377

**Published:** 2012-07-09

**Authors:** Josef Thingnes, Timothy J. Lavelle, Eivind Hovig, Stig W. Omholt

**Affiliations:** 1 Centre for Integrative Genetics (CIGENE), Department of Mathematical Sciences and Technology, Norwegian University of Life Sciences, Ås, Norway; 2 Department of Tumor Biology, Institute for Cancer Research, The Norwegian Radium Hospital, Oslo, Norway; 3 Institute of Informatics, The Faculty of Mathematics and Natural Sciences, University of Oslo, Oslo, Norway; 4 Institute of Medical Informatics, The Norwegian Radium Hospital, Oslo, Norway; 5 Centre for Integrative Genetics (CIGENE), Department of Animal and Aquacultural Sciences, Norwegian University of Life Sciences, Ås, Norway; University of Tennessee, United States of America

## Abstract

The strikingly even color of human skin is maintained by the uniform distribution of melanocytes among keratinocytes in the basal layer of the human epidermis. In this work, we investigated three possible hypotheses on the mechanism by which the melanocytes and keratinocytes organize themselves to generate this pattern. We let the melanocyte migration be aided by (1) negative chemotaxis due to a substance produced by the melanocytes themselves, or (2) positive chemotaxis due to a substance produced by keratinocytes lacking direct physical contact with a melanocyte, or (3) positive chemotaxis due to a substance produced by keratinocytes in a distance-to-melanocytes dependent manner. The three hypotheses were implemented in an agent-based computational model of cellular interactions in the basal layer of the human epidermis. We found that they generate mutually exclusive predictions that can be tested by existing experimental protocols. This model forms a basis for further understanding of the communication between melanocytes and other skin cells in skin homeostasis.

## Introduction

Coloration of human skin is due to melanin pigments that are produced by melanocytes in the basal layer of the epidermis. Each melanocyte attached to the epidermal basement membrane exports mature melanosomes to nearby keratinocytes through its dendrites. The uptake of melanosomes by the keratinocytes is an active process involving the dendrites and filopodia of the melanocyte, as well as regulatory processes in the keratinocytes [1], [2]. 95% of the cells in the epidermis are keratinocytes and a fraction of the keratinocytes in the basal layer are “stem” keratinocytes, which produce new keratinocytes continuously through cell division. From being attached to the epidermal basement membrane initially, the “non-stem” keratinocytes move progressively upwards and make up the upper cell layers of the epidermis [3], [4].

The skin color in young and healthy individuals is remarkably uniform. A major reason for this is that the melanocytes are evenly distributed throughout the basal layer of the epidermis [5]. The density of melanocytes varies with the body site from around 900 melanocytes per square mm on the back to around 1500 melanocytes per square mm in the genital region [4]. Comparing the same body site, the individual variation is remarkably small, even when comparing skin of differing complexion [6], [7]. The uniform melanocyte distribution is maintained despite varying melanocyte densities between body sites, and is restored after temporal destruction of melanocytes due to UV-light overexposure or moderate wound infliction [3], [7], [8], [9], [10], [11]. The existence of an underlying dynamic regulatory scheme responsible for this maintenance through influence on melanocyte proliferation and/or migration is thus most likely. Additional support for this comes from a study where a cell slurry containing human keratinocytes, fibroblasts and melanocytes was poured into silicone chambers implanted directly on the muscle fascia of severe combined immunodeficient mice [12]. The cells spontaneously reorganized into functioning dermis and epidermis with the melanocytes contained in the basal layer of the epidermis. This human skin substitute was uniformly colored with a complexion comparable to the one of the melanocyte donor [12].

The concept of one melanocyte interacting specifically with a specified group of keratinocytes was first proposed by Fitzpatrick and Breathnach [13] in 1963. They proposed that “the epidermal melanin unit” consist of one melanocyte and approximately 36 keratinocytes. The study of melanocyte density can be viewed as the study of the size of the epidermal melanin unit. Scott and Haake [14] conducted an experiment in 1991 where they constructed skin equivalents from neonatal and fetal melanocytes and keratinocytes and showed that the keratinocyte was the key determinant of the size of the epidermal melanin unit in that model. Also skin explants have been used in the study of melanocyte density and migration; Le Poole [15] showed in 1994 how melanocytes proliferated and migrated to populate newly formed epibolic outgrowth of keratinocytes.

The major focus of this theoretical study is to contribute to the elucidation of the mechanisms by which melanocytes maintain an even distribution in the basal layer of the epidermis by migration, proliferation and cell death. Further, this model will form a basis for further efforts to understand this communication in development, homeostasis, wound healing, and malignant transformation. The mathematical conceptualization of the biology involved can be done in several ways. Because the characteristic even distribution of melanocytes is likely to be an emergent property of the ‘decisions’ made by the individual cells in the epidermis, based on cues in their immediate environment and their own genetic and epigenetic constitution, we anticipated that an individual-based modeling framework would be appropriate for this type of problem. Among the individual-based modeling platforms available, we chose the general-purpose agent-based modeling framework FLAME (Flexible Large-scale Agent-based Modeling Environment, http://www.flame.ac.uk/). The major reason for this choice was that FLAME was developed in close interaction with groups making computational models of epithelial tissue [16].

Our model of the dynamics within the human epidermis basal layer takes advantage of two models already developed within the FLAME environment, one describing in vitro keratinocyte colony formation [17] and one describing how fibroblasts support this keratinocyte colony formation [18]. These models are based on the transit amplifying/stem keratinocyte model of epidermal turnover, reviewed in [19]. From these two models, we chose rules to emulate keratinocytes growing on a basal membrane with an intact dermis (or dermal equivalent) underneath. We then extended this combined model by incorporating melanocytes, associated behavioral rules and the diffusion of regulatory signals.

With this model framework, we tested whether the uniform distribution of melanocytes could be considered to be a self-organization phenomenon based on local chemical cues. More specifically, is the behavioral response of the individual melanocyte to a local chemical gradient of one or more molecules produced either by melanocytes or keratinocytes sufficient to explain the observed uniformity? The skin is in possession of a vast arsenal of both ligands and receptors that can be involved in such a mechanism [20], [21]. To this end, different mechanisms may be hypothesized. First, since the melanocyte has been proposed to harbor both sensory and regulatory properties [22], one may anticipate a repellent signal R produced by the melanocytes which causes melanocytes to stay away from each other. All melanocytes will migrate down the gradient of R and the concentration of R will determine if the melanocyte would proliferate or die. Candidates for this substance are the intermediate products in the melanin synthesis; L-tyrosine and L-DOPA [23]. Second, one may also envisage an attracting signal A made by keratinocytes in need of melanocyte contact, causing positive chemotaxis. The simplest form of this mechanism is a constant production of the attracting signal in all keratinocytes not having a melanocyte in its immediate neighborhood. This binary production condition maps well to the hypothesis that melanocyte dendrites are connected to keratinocytes and deliver their melanosomes directly to the cytosol of the keratinocytes (discussed in [24]). Third, it is also plausible that a positive chemotaxis scheme may be set up by the production of an attractant A by keratinocytes, where the production rate is continuously dependent on the distance to surrounding melanocytes or the degree of physical contact.

The agent-based model successfully predicts the observed uniformity of melanocyte distribution for a range of melanocyte densities for each of the three proposed mechanisms. However, we show that the putative mechanisms generate partially mutually exclusive testable predictions. By incorporating the dynamics of both keratinocytes and melanocytes, the model may provide a point of departure for addressing underlying mechanisms regulating melanocyte re-invasion and maintenance of homeostasis in the human epidermal basal layer.

### Outline of the Model

An agent-based model has two parts; the environment and the agents. The environment consists in this case of only the physical space that restricts the movement of the agents *i.e.* the virtual dish. The agents represent cells of three different types; stem keratinocyte, transit amplifying (TA) keratinocyte and melanocyte. In all simulations in this work, the virtual dish is a square with sides 400 µm. To avoid observing artifacts from our boundary conditions, all observations were done in a 300 µm by 300 µm square located 50 µm from all edges. The cells in the virtual dish are each represented by an individual agent. The agents communicate (cell signaling) by writing to and reading from message lists. The model describes the dynamics in 30 minutes steps. The agents go through a defined sequence of procedures in each iteration. Initially, agents (cells) output their location and type (stem keratinocyte, TA keratinocyte, or melanocyte) to the message lists for other cells to read. All agents maintain two variables, containing a quantitative measure of the strength of signal R and/or signal A at the cell’s location, which may reflect signal propagation or signal substance diffusion from cell to cell. To update the levels of signal substances, all cells are required to complete particular steps before any cell can continue. These steps include updating the amount according to production rate, updating levels according to substance leaving the cell because of diffusion, and finally outputting this information for the neighbors to read. When all cells have written their out-going diffusion amount, each cell can interrogate the message lists to find the information needed for updating the substance levels according to the influx of substances from its neighbors. Next, the degradation of signal substance is accounted for, before the signaling substance module is completed, by the posting of the current levels to the message lists (for further use by the migration step). Each cell then applies cell cycle propagation rules specific to its own cell type and position in the cell cycle, which may conclude in cell division or cell death. The progression of the cells in the cell cycle is based on availability of space and differentiation state. The melanocytes will, in addition, rely on the strength of the two signals R and/or A for its cell cycle progression. Following this, the stem keratinocytes decide whether to change to TA keratinocytes based on the differentiation rules in the model. Cells then execute their migration. While stem keratinocytes are tightly bound to the substrate and rather stationary, the TA keratinocytes have, to some extent, a random migration pattern [17], [25]. Melanocytes on the other hand, migrate according to the gradient of signal in its immediate neighborhood. The direction of migration is determined by the position of two neighboring cells; the one with the weakest and the one with the strongest signal. The magnitude of the migration is determined by the difference between the signal strength of these two neighbors. As the actions of cell migration and division are modeled as discrete steps that are applied to each agent individually, it is possible for the simulated cells to overlap in their virtual culture dish. In this instance, a corrective repulsive force is applied in order to push the cells apart. This is proportional to the overlapping area (a higher force for a bigger overlap). Attractive forces simulate bonds between cells and the substrate, and are applied when the respective bodies (cell–cell or cell–substrate) are within 10 µm of one another. All rules are executed in the context of the agent’s own internal state and its immediate environment as discovered through interrogation of the message lists.

## Results

### Two of the Three Mechanisms are able to set up Global Gradients of Signal Strength

**Figure 1:**
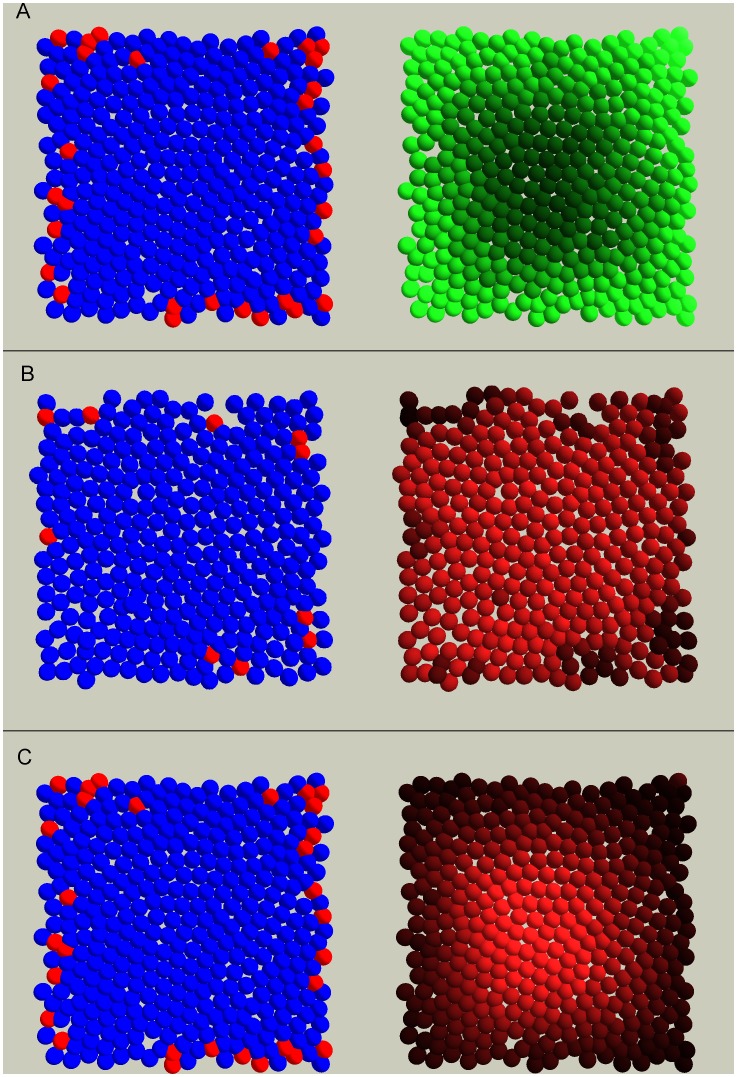
Signal gradient as produced by our diffusion model. Two different views of three different virtual cell cultures are shown. In the left panels the cells are colored according to cell type; keratinocytes blue and melanocytes red, while in the right panels cells are colored according to strength of signal (lighter color equals higher concentration). The signal substance diffuses from cell to cell and degrades according to our diffusion model. In these simulations, we have restricted the melanocytes to reside at the outer rim of the dish in order to visualize the global gradient towards the middle. In A, the cells are colored according to the concentration of signal substance R that is produced by all melanocytes at a constant rate. In B and C, the cells are colored according to the concentration of the attracting signal substance A. The gradient in B is generated by production of signal in all keratinocytes not in contact with a melanocyte (A, binary), while in C the gradient is set up by production of signal in all keratinocytes as a function of the strength of the signal R (A, R-dependent).

In order to visualize the ability of the three mechanisms to set up global gradients, we performed simulations where we restricted the melanocytes to the rim of the virtual dish and colored the virtual cells according to their signal strength (**Figure 1**). The repellent R, produced by the melanocytes, displays a gradient with high concentration around the edges and lower concentrations in towards the middle. The gradient of the attractant A differs substantially between the binary and the continuous production regimes. In the binary case, where there is production of substance A at a uniform level in all keratinocytes not in contact with melanocytes, no global gradient is visible but rather a uniform signal strength, except for patches of lower concentrations around each melanocyte. On the other hand, in the continuous case, where the production of substance A depends on the local concentration of signal R, there is, as would be expected, a close to inverted image of the gradient set up by R.

### The Proposed Mechanisms are all Sufficient to Produce the Observed Even Melanocyte Distribution for Melanocyte Densities 5% and 10%

**Figure 2:**
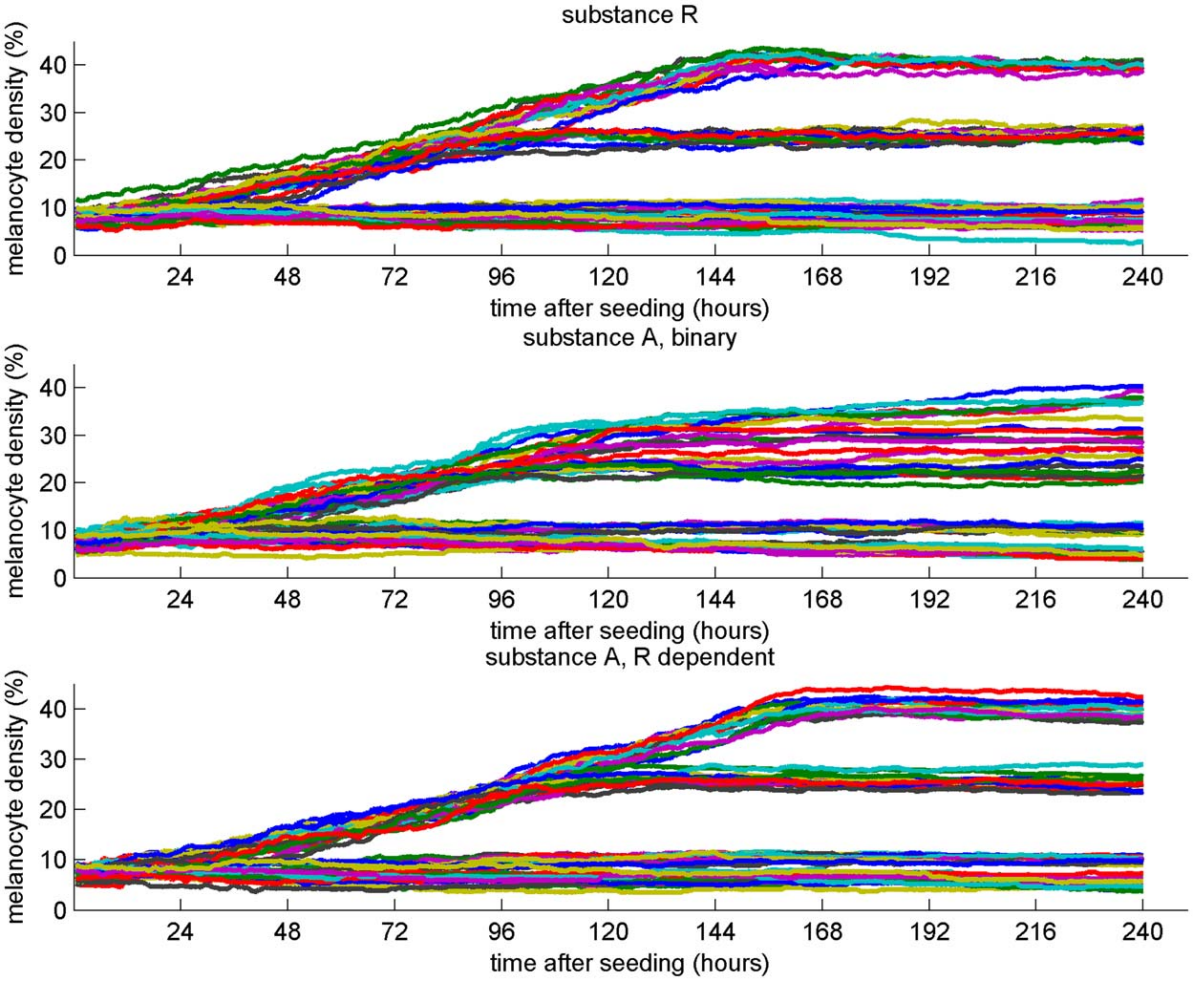
Melanocyte density under different parameter settings. The three mechanisms for guiding melanocyte growth and migration were tested for their ability to establish a uniform melanocyte distribution at 5%, 10%, 25%, and 40% melanocyte densities. The temporal developments of the melanocyte density throughout the 10 days of simulation are shown. The simulations were repeated 12 times for each of the four parameter settings.

**Figure 3:**
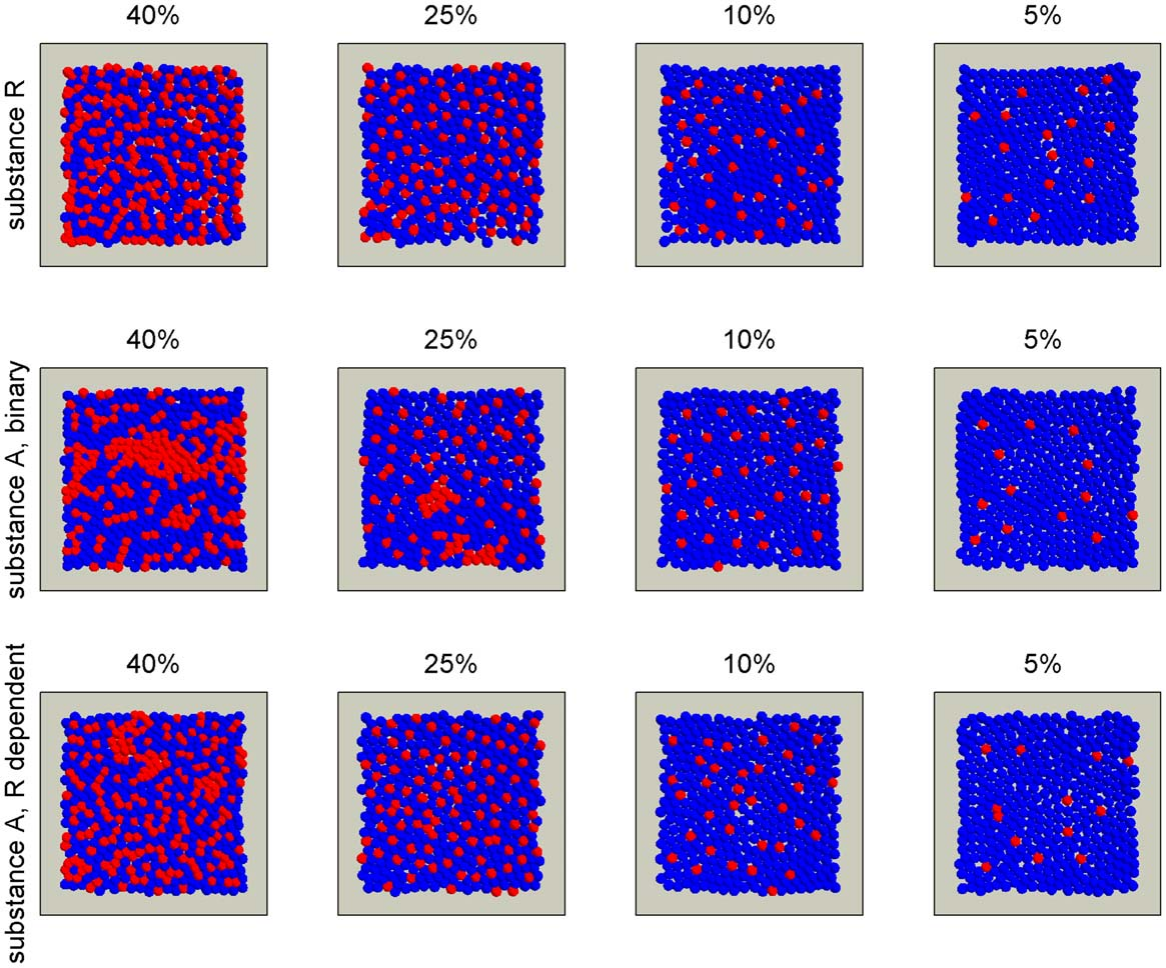
The observation area of the virtual dish after ended simulations. Simulations were performed with parameters set to establish four different melanocyte densities as indicated, for all three mechanisms proposed. All simulations were performed in 12 replicates; one representative image is shown for each parameter setting and mechanism.

The melanocyte density in the human epidermal basal layer is most often referred to as 5–10% [3], [26]. But by comparing melanocyte counts and statements about cell dimensions, it seems likely that the density of melanocytes can be substantially higher [3], [7], [8], [9], [10], [11]. In embryos, as much as 2301±41 melanocytes per square mm have been observed [5]. In chronically sun-exposed skin, melanocytes in a ratio of one melanocyte for every tree keratinocyte have been observed [27]. The mechanism responsible for the melanocyte distribution must be able to function within this observed range. To make sure that we covered the observed ranges of densities, we simulated melanocyte densities in the range 5–40%. In order to test how well the three proposed mechanisms were able to produce evenly distributed melanocyte patterns within the entire range, we simulated three cell culture experiments lasting 10 days. For each experiment, parameters were set to establish melanocyte densities at 5%, 10%, 25%, and 40%, and the simulations were repeated 12 times for each parameter setting (**Figure 2** and **Figure 3**). Both the repellent and the continuously produced attractant were able to establish uniform melanocyte distributions at all densities. The binary attractant production regime broke down at the highest densities. This is as expected since at uniform melanocyte densities above 15% - 20% all keratinocytes will have direct contact with a melanocyte and therefore no guiding signal will be produced. At the lower densities (5% and 10%), all three mechanisms seem able to establish uniform melanocyte distributions. See Video S 1, Video S 2, and Video S 3 for example movies of the virtual dish.

### Mutual Exclusive Lab Testable Predictions

**Figure 4:**
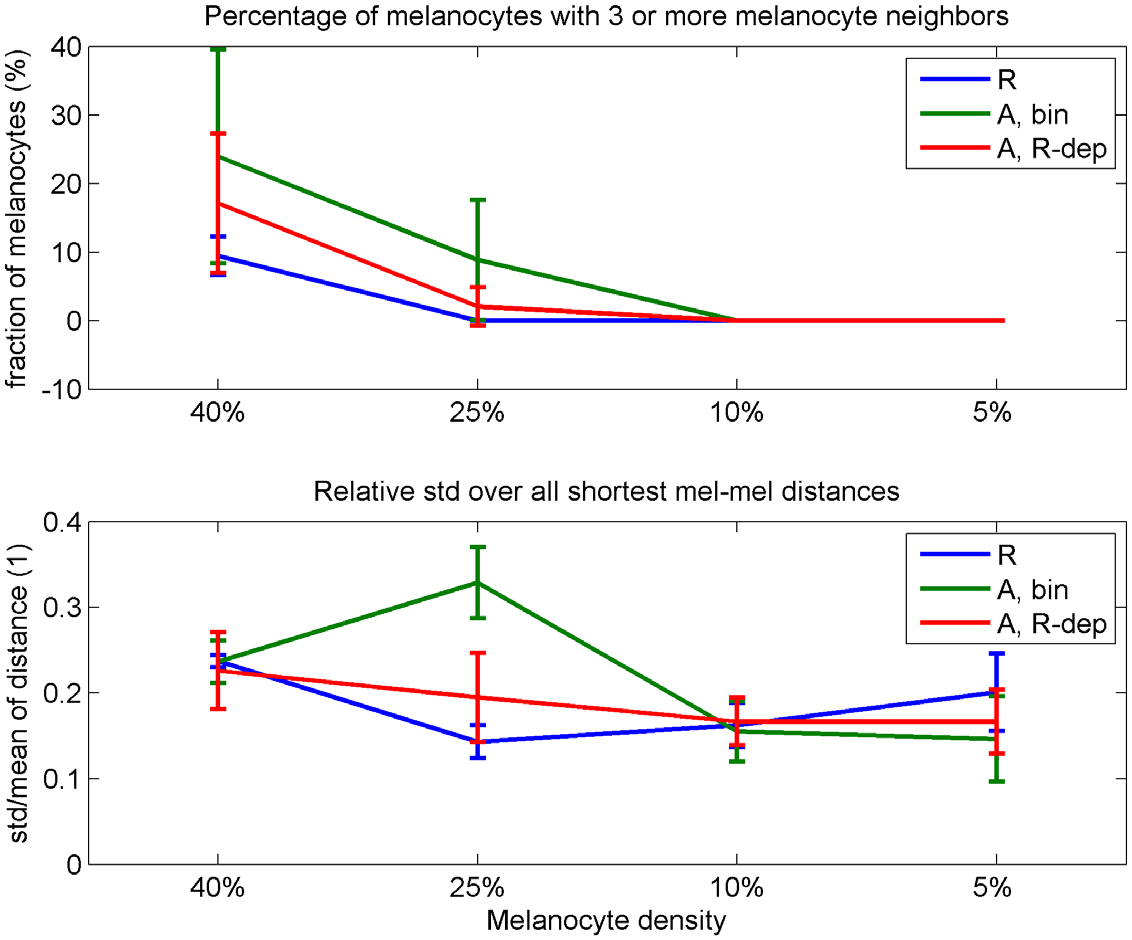
Melanocyte uniformity measurements. As quantitative measurements of the three different mechanisms’ ability to distribute melanocytes evenly, we counted the number of melanocytes with three or more melanocyte neighbors (top), and the relative standard deviation of the distance from all melanocytes to the nearest other melanocyte (bottom). All measurements are given as mean and standard deviation of 12 repetitions. A Kolmogorov–Smirnov test was performed to test for significant differences between the hypotheses at each parameter setting of which the results are given in **Table 1**.

As quantitative measures of the evenness of the melanocyte distribution, we calculated the percentage of melanocytes with more than three melanocyte neighbors, as well as the relative standard deviation of the shortest distance from each melanocyte to another melanocyte at the end of the 10 days experiment (**Figure 4**). The first measure did not disclose different predictions of the three hypotheses, except at the highest densities (40%). The second measure picked up distinct predictions for each hypothesis at 25% melanocyte density. In addition, the three hypotheses generated qualitatively different profiles in the standard deviation plot between 5% and 25% melanocyte density. While the two attractant approaches have a relative standard deviation of shortest distances between melanocytes that increases with higher melanocyte density, the repellent approach predicts a decreasing trend.

## Discussion

We have here presented predictions containing properties that may be tested in carefully designed lab experiments for validation of the mechanisms proposed herein. For this computational model to become a coherent predictive tool, the parameters concerning the melanocytes migration, cell cycle progression and apoptosis need to be more accurately determined. The key experiment that could help pin down several of these parameters is one where a human skin equivalent is produced either *in vitro* or using a mouse system, where both the melanocytes and the keratinocytes in the basal layer can be tracked. Further, it would be of great value to be able to measure levels of paracrine signals in such a model tissue using time series observations.

The overall impression of the three mechanisms’ performance is that the binary regime producing the attractant A performs less well than the two alternatives. However, this is valid only for the two highest melanocyte densities. For 5% and 10% melanocyte densities, the binary production regime is able to produce plausible melanocyte distributions (**Figure 2** and **Figure 3**) despite its poor ability to set up a global gradient (**Figure 1**).

Most reports on melanocyte densities are given in absolute number of melanocytes per length unit (of a cross section) or absolute number of melanocytes per area unit [3], [7], [8], [9], [10], [11]. The density relative to melanocyte numbers thus has to be deduced. A biopsy study revealing the relative melanocyte density in the human epidermis across skin colors and body sites would be of great value.

This model may be developed further to also describe the melanin delivery process, and can thus become instrumental for the understanding of how melanin in different layers of the epidermis is distributed in differently colored skin types. Moreover, this model can serve as scaffolding for the development of a quantitative understanding of the biological mechanisms of freckles, and moles.

## Methods

In the following, a brief introduction to the agent-based modeling approach is given in addition to explanations to the specific features of the model used herein. For a deeper introduction to the theoretical background of agent-based modeling see [16], [28] and for a more detailed description of the modeling tool FLAME, see previously published models *e.g.* [29], [30], [31], [32], [33]. The concept of this melanocyte-keratinocyte co-culture model is based on two earlier published models; a keratinocyte colony formation model [17] and a normal human keratinocyte – human dermal fibroblast co-culture model [18]. In the present work, we have developed a model that mimics melanocyte-keratinocyte co-culture growing on top of a basement membrane with a functioning dermis or dermal equivalent. To achieve this, the keratinocyte colony formation model was extended with rules from the keratinocyte/fibroblast co-culture to mimic the microenvironment set up by the fibroblasts in the dermis. We anticipated that all keratinocytes in the basal layer be sufficiently fed, as well as obtaining the fibroblast contact needed for normal growth and survival.

**Figure 5:**
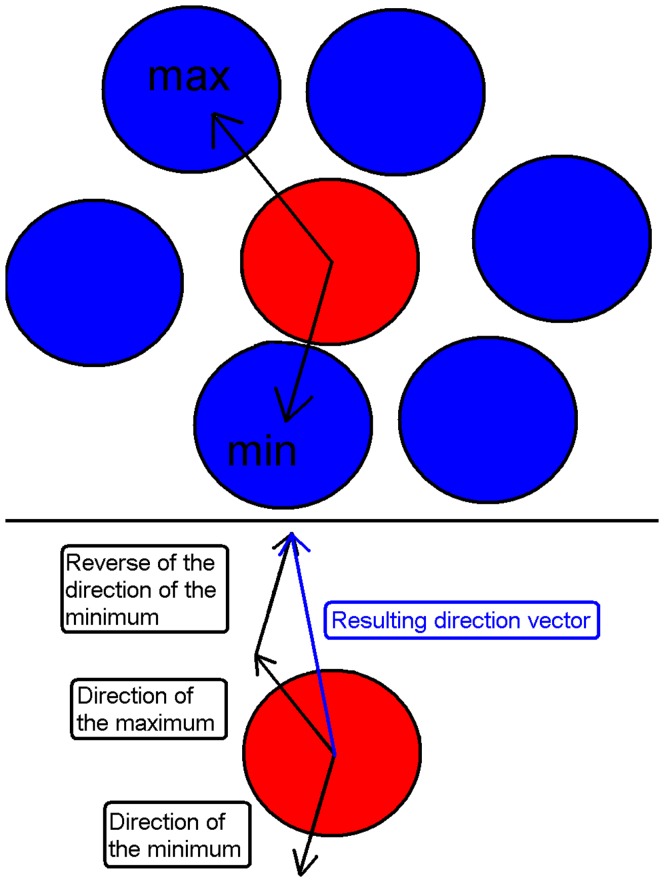
Algorithm for calculation of the migration direction. The direction of the maximum and minimum substance values in the neighborhood is recorded. The resulting migration vector is a sum of two vectors: One with direction towards the maximum substance level and one in the opposite direction of the minimum substance level.

Each cell was modeled as a non-deformable sphere (20 µm in diameter) governed by a rule set, and cells were capable of migration, proliferation and differentiation. In this study, the culture dish was modeled as a flat square surface (400 µm×400 µm) with a wall around it (**Figure 1**). To avoid observing artifacts from our boundary conditions, all observations were done in a 300 µm by 300 µm square located 50 µm from all edges. As the cells stratified to form a three dimensional skin equivalent, they were deleted from the model. Even if signals from the suprabasal layers of epidermis probably are important for the determination and formation of the epidermal melanin unit, the introduction of the third dimension would not affect the hypothesis tested herein. Thus, this is a justifiable measure to diminish CPU-time. The following is the agent rule sequence. Initially, agents (cells) output their location and type (stem keratinocyte, transit amplifying (TA) keratinocyte, or melanocyte) to the message lists for other cells to read. To mimic the two signals investigated in this study, all agents maintain two variables containing a quantitative measure of the strength of signal R and signal A in the tissue at the location of the cell. The dynamics of these variables could represent signal propagation or signal substance diffusion from cell to cell. To update the levels of signal substances, all cells have to finish particular steps before any cell can continue. These steps are; updating the amount according to production rate, updating levels according to substance leaving the cell because of diffusion and finally, output this amount for the neighbors to read. When all cells have written their out-going diffusion amount, each cell can interrogate the message lists to find the information needed for updating the substance levels according to the influx of substances from its neighbors. Next, the degradation of signal substance is accounted for, before the signaling substance module is completed by posting of the current levels to the message lists (for further use by the migration step). Each cell then applies cell cycle propagation rules specific to its own cell type and position in the cell cycle, which may conclude in a cell division or cell death. The advancement of the cells in the cell cycle is based on availability of space (contact inhibition) and differentiation state (the latter only for keratinocytes), as is observed for epidermal cells [15]. The melanocytes will, in addition, rely on the strength of the two signals R and A for its cell cycle progression. This concept is implemented by an integer variable that is incremented if the conditions are satisfied. When the value in this variable reaches a threshold, the cell divides and a new agent is created. Following this, the stem keratinocytes decide whether to change to TA keratinocytes based on the differentiation rules in the model. Cells then execute their migration. While stem keratinocytes are tightly bound to the substrate and rather stationary, the TA keratinocytes have, to some extent, a random migration pattern. Melanocytes on the other hand, migrate according to the gradient of signal in its immediate neighborhood. The migration direction is determined by the position of two neighboring cells; the one with the weakest and the one with the strongest signal (**Figure 5**). The migration is parallel to the line between these two cells and the direction along the line is determined by positive or negative chemotaxis. The magnitude of the migration is determined by the difference between these two neighbors’ signal strength. As the actions of cell migration and division are modeled as discrete steps that are applied to each agent individually, it is possible for the simulated cells to overlap in their virtual culture dish. In this instance, a corrective repulsive force is applied in order to push the cells apart. This is proportional to the overlapping area (a higher force for a bigger overlap). Attractive forces simulate bonds between cells and the substrate, and are applied when the respective bodies (cell–cell or cell–substrate) are within 10 µm of one another. All rules are executed in the context of the agent’s own internal state and its immediate environment, as discovered through interrogation of the message lists. The framework with a detailed user manual is freely available for users to download (FLAME, http://www. flame.ac.uk).

### Production Rate of Substance R

The repellent substance R is produced by melanocytes to signal to other melanocytes. The idea is to investigate the possibility of such a substance. This substance can be any signal substance produced by the melanocyte, but it can also be viewed as the melanin. We have set the production rate to one constant level in all melanocytes.

### Production Rate of Substance A

The substance A represents any signal produced by the keratinocytes to attract melanocytes. In this work, two production regimes of substance A are investigated. Under the binary regime, the keratinocytes produce substance A at a constant level if they lack a melanocyte in their immediate neighborhood and shut down the production if the melanocyte contact is present. In the more sophisticated regime, the signal A is produced as a function of the distance to the neighboring melanocytes. As a measure of this distance, we have used the signal R sent by melanocytes. In this regime, the signal R does not work as a repellant, but only as a measure of distance to surrounding melanocytes. The substance A production is negatively dependent on the concentration of R and follows this equation:
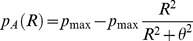
(0.1)


Where θ is the level of substance R where the production 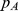
equals half its maximal production 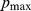.

### A Model of Diffusion and Degradation

A common way to model diffusion and degradation is by a partial differential equation (PDE). To numerically solve the PDE, the space of interest is divided into compartments where the dynamics of the concentration is managed by an ordinary differential equation (ODE). The compartments must be small enough to give the desired resolution, but still few enough to make the computation feasible. To simulate the density of small signaling molecules that travel from cell to cell through gap junctions, we found the keratinocytes and melanocytes themselves to be the most suitable compartments. In this way, the signal substance distribution effect of several mechanisms was included:

- The depletion effect of proliferation
- The depletion effect of keratinocytes moving upwards and out of the monolayer
- The distribution effect of a migrating cell carrying a particular concentration of the signal molecules
- The effect of the moving sources (the keratinocytes and melanocytes)

In the setting of this agent-based model, the key equation describes the strength of signal (or amount of signal substance) in each cell at the next iteration, in terms of information about signal strength and cell positions in the current iteration. The strength 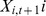 of a signal at time step 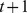 in cell 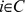 can be described in terms of the signal strength 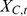 and position 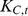 in all cells 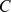 at time 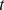:
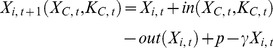
(0.2)where 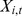 is the signal strength in cell 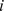 at time 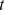, the function 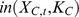 describes the influx of signal from the neighboring cells, the function 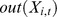 describes the outflux of signal to neighboring cells, 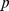 is the local production according to any of the production regimes described above, and 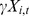 is the degradation. The outflux is modeled to be a constant fraction of the current amount, and this fraction is shared equally between the neighbors. The neighbors of a cell are defined as the set of cells being nearer than a threshold distance. The influx 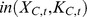 is the sum of all the outgoing shares bound for this cell originating in the neighboring cells.

### Melanocyte Motility

Melanocytes migrate up the gradient of the attracting substance A or down the gradient of the repelling substance R. Cell migration in 2D is usually modeled as a series of events where the cell attaches to the substrate (in our case the basement membrane) on the side which faces the direction to which it ‘wants’ to go and releases attachments on the side facing the direction it ‘wants’ to leave. Cell motility in general has been modeled for centuries, and is reviewed in [34]. On the basis of this concept, we have made a simple algorithm for melanocyte migration. The algorithm can be written in pseudo-code like the following:

For all neighbors i


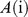
  =  neighbor(i) -> substance_A_level


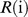
  =  neighbor(i) -> substance_R_level


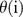
  =  The direction from the current cell to neighbor(i)

End
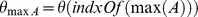


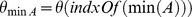


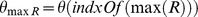


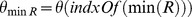


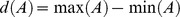


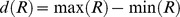



If leaded by substance A
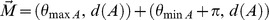



Else
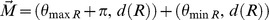



End

**Table 1:** Strength of exclusivity: Kolmogorov–Smirnov test on the data plotted in Figure 4.

Neighbor count	40%	25%	10%	5%
R/A-bin	**	**		
R/A-cont	**	**		
A-bin/A-cont				
Relative standard deviation	40%	25%	10%	5%
R/A-bin		**		*
R/A-cont		*		
A-bin/A-cont		**		

For each of the two measures of uniformity, the significance of difference between each pair of hypothesis is reported. Abbreviations used: R: the hypothesis that the melanocytes are guided by a repellent, A-bin: the hypothesis that the melanocytes are guided by an attractant under a binary production regime, A-cont: the hypothesis that the melanocytes are guided by an attractant under a continuous production regime. The Null-hypothesis that the two data sets are drawn from the same distribution is rejected where indicated. *: P<0.05, **: P<0.01.

where 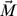 is the total migration vector (all vectors are on the form 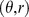 where 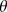 is the angle and 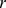 is the radius). Using words, the algorithm can be described like this: The movement of a melanocyte is the sum of two vectors. Melanocytes move up the substance A gradient or down the substance R gradient. In practice, that means away from the low density of substance A or towards the high density of substance A, which represents attaching to the substrate on the side with high density and obliterating attachment at the side with low density. This is implemented as two movements with length proportional to the difference between the maximal and minimal substance A concentration in the neighborhood, one in the direction of the highest concentration and one in the opposite direction of the lowest concentration. The same is done for substance R, although in the opposite direction, since the melanocytes are moving down that gradient (Figure 5). This way of reacting to the very local gradient may lead to a weighted random walk, which is in accordance with observations for cell migration on gradients [35].

### The Quantitative Measures of the Evenness of the Melanocyte Distribution

The ratio of the melanocytes that have more than three melanocyte neighbors was calculated. For two melanocytes to be counted as neighbors, they have to be in physical contact (i.e. the center to center distance is less than or equal to the cell diameter, 20 µm). Only the melanocytes within the 300 µm by 300 µm observation area are included in the calculation. However, when counting neighbors, all melanocytes are counted, even those residing outside the observation area. For each repetition, for each parameter setting, for each hypothesis, the total number of melanocytes and the number of melanocytes with more than three neighbors was measured. For both measurements, the mean of the last 48 iterations (24 h) were used as the basis for the ratio reported in **Figure 4**. In **Figure 4**, the mean and standard deviation over the 12 repetitions are reported for each hypothesis and parameter setting. The Kolmogorov–Smirnov test results reported in **Table 1** are obtained by applying the MATLAB function “kstest” as implemented in MATLAB version 7.2.0.232 (R2006a).

The relative standard deviation of the shortest distance from each melanocyte to another melanocyte at the end of the 10 days experiment was calculated. Only the melanocytes within the 300 µm by 300 µm observation area are included in the calculation. However, when calculating the distance to the nearest melanocytes, all melanocytes are considered, even those residing outside the observation area. For each melanocyte within the observation area, the shortest distance to another melanocyte was monitored and the mean (mdist) and standard deviation (sddist) over these distances saved for each iteration. For each repetition, for each parameter setting, for each hypothesis, the mean of the last 48 iterations (24 h) of sddist was divided by the mean of the last 48 iterations (24 h) of the mdist to form the relative standard deviation (relsd). In **Figure 4**, the mean and standard deviation of relsd over the 12 repetitions are reported for each hypothesis and parameter setting. The Kolmogorov–Smirnov test results reported in **Table 1** are obtained by applying the MATLAB function “kstest” as implemented in MATLAB version 7.2.0.232 (R2006a).

## Supporting Information

Video S1 **Melanocytes guided by repellent substance, 10% melanocyte density.**
(WMV)

Video S2 **Melanocytes guided by attractant substance under binary production regime, 10% melanocyte density.**
(WMV)

Video S3 **Melanocytes guided by attractant substance under continuous production regime, 10% melanocyte density.**
(WMV)
